# Limb dominance influences landing mechanics and neuromuscular control during drop vertical jump in patients with ACL reconstruction

**DOI:** 10.3389/fphys.2024.1488001

**Published:** 2024-10-21

**Authors:** Boshi Xue, Xiaowei Yang, Xia Wang, Chen Yang, Zhipeng Zhou

**Affiliations:** ^1^ College of Sports and Health, Shandong Sport University, Jinan, China; ^2^ Faculty of Sports Science, Ningbo University, Ningbo, China; ^3^ College of Sports and Health, Nanjing Sport Institute, Nanjing, China

**Keywords:** ACLR, landing strategy, landing mechanics, muscle activation, return to sport

## Abstract

**Objectives:**

The purpose of this study was to compare the interlimb biomechanical differences in patients who had undergone anterior cruciate ligament reconstruction (ACLR) in either dominant (ACLR-D) or nondominant (ACLR-ND) limbs and healthy controls (CON) during drop vertical jump (DVJ) task. To investigate whether the dominant or nondominant limb influences the risk of re-injury in ACLR patients.

**Methods:**

Thirty-three ACLR patients were divided into ACLR-D and ACLR-ND groups according to whether the surgical limb was dominant or nondominant. Seventeen healthy individuals were selected as the CON group. Three-dimensional kinematic data, ground reaction force (GRF) data, and surface electromyographic (EMG) data from the bilateral lower limbs of all participants were collected during the DVJ task. Two-way repeated-measures ANOVAs (limb × group) were performed on the variables of interest to examine the main effects of limb (dominant vs. nondominant) and group (ACLR-D, ACLR-ND, and CON), as well as the interaction between limb and group.

**Results:**

The nonsurgical limbs of ACLR group had significantly greater knee valgus angles, knee extension and valgus moments, peak posterior GRF (PPGRF), and peak vertical GRF (PVGRF) compared to the surgical limbs. The nonsurgical limbs of ACLR-ND patients demonstrated significantly greater knee extension and valgus moments, greater PPGRF and PVGRF, and reduced muscle activity in the vastus medialis and vastus lateralis compared to the CON group. The ACLR patients had reduced muscle activity in the quadriceps of the surgical limb and the hamstrings of the bilateral limbs compared to controls.

**Conclusion:**

The nonsurgical limbs of ACLR patients may suffer an increased risk of ACL injury due to altered landing mechanics and neuromuscular control strategies compared to the surgical limbs. Additionally, limb dominance influences movement patterns and neuromuscular control during DVJ task, the nonsurgical limbs of the ACLR-ND might be at higher risk of ACL injury compared to the ACLR-D group.

## 1 Introduction

Anterior cruciate ligament (ACL) injuries are among the most prevalent severe sports injuries, accounting for approximately 50% of all knee injuries (Moses et al., 2012; Kaeding et al., 2017). Following ACL injuries, patients experience abnormal neuromuscular control, decreased knee stability, and increased risk of knee osteoarthritis (Howells et al., 2011; Gersing et al., 2021). ACL reconstruction (ACLR) is a common surgical treatment following ACL injuries, contributing to restoring knee function and safely returning to play. However, a quarter of young athletic patients suffered an ACL re-injury (Wiggins et al., 2016), suggesting significantly higher injury rates compared to primary ACL injuries. The incidence rates of the surgical limb and nonsurgical limb were reported as 7%–12% and 18%–28%, respectively (Webster and Feller, 2016; Lindanger et al., 2019). Therefore, it is critical to monitor the rehabilitation progress on both surgical and nonsurgical limbs following ACLR.

Bilateral asymmetries in knee mechanics during landing were commonly observed following ACLR (Johnston et al., 2018; King et al., 2021; Kotsifaki et al., 2022b; Kotsifaki et al., 2022a), which has been considered as ACL reinjury risk factors (Johnston et al., 2018; King et al., 2021). The surgical limbs typically exhibit smaller knee flexion angles, knee extension moments, and ground reaction force (GRF) during landing (Johnston et al., 2018; Kotsifaki et al., 2022a), whereas greater knee joint contact forces and ACL forces are present in the nonsurgical limbs (Wren et al., 2018; Rush et al., 2024). In fact, the limb dominance may be associated with bilateral asymmetry in health populations during jump task (Edwards et al., 2012). Abnormal landing kinematics and kinetics for dominant and nondominant limbs, including greater valgus angles and peak GRF for nondominant limbs during jumps (Wollschläger-Tigges and Simpson, 2016; Nakahira et al., 2022), as well as higher knee extension moments and quadriceps activation for dominant limbs (Yılmaz and Kabadayı, 2022), may contribute to increased risk of ACL injuries. Therefore, whether the limb dominance contributes to the bilateral asymmetry in ACLR patients need to be investigated.

In fact, recent work reported the bilateral biomechanical characteristics in relation to the limb dominance following ACLR; however, the results for dominant and nondominant are inconclusive (Dos’ Santos et al., 2019; Malafronte et al., 2021; Farmer et al., 2022; Goto et al., 2022). A recent study showed that for the surgical limb, patients underwent ACLR on the nondominant limb had greater knee loading (peak knee extension moments, peak patellofemoral joint stresses) during walking compared to patients underwent ACLR on the dominant limb (Goto et al., 2022). Conversely, Malafronte et al. (2021) reported that patients with ACLR on the dominant limb demonstrated greater knee joint loading in surgical limb compared to nondominant ACLR during jump-landing task. Meanwhile, for nonsurgical limbs, the results between dominant and nondominant limbs seem to be contradictory. Goto et al. (2022) found that dominant ACLR patients carried 49% more knee load in walking than nondominant ACLR patients. However, Malafronte et al. demonstrated that dominant ACLR patients carried 76% less knee load than nondominant ACLR patients performing jump-landing task (Malafronte et al., 2021). The above results suggest that there may be biomechanical differences between dominant and nondominant limbs in patients with ACLR, but the findings regarding the risk of secondary ACL injury or graft rupture in the surgical and nonsurgical limbs are inconsistent across studies.

The purpose of this study was to compare the biomechanical characteristics of bilateral limbs in patients who had undergone ACLR in either dominant (ACLR-D) or nondominant (ACLR-ND) limbs and healthy controls (CON) during drop vertical jump (DVJ) task. We hypothesized that (1) the nonsurgical limbs would exhibit smaller knee flexion angles, greater GRFs, greater knee extension and valgus moments, and greater quadriceps and hamstring muscle activation compared to the surgical limbs, regardless of ACLR-D or ACLR-ND group, and (2) the nonsurgical limbs in the ACLR-ND group would exhibit smaller knee flexion angles, greater knee extension and valgus moments, greater GRFs, and greater quadriceps and hamstring muscle activation compared to the nonsurgical limbs in the ACLR-D group.

## 2 Material and methods

### 2.1 Participants

Based on an estimated effect size of 0.78 for differences in knee extension moments between limbs of the ACL-D and ACL-ND (Goto et al., 2022), a sample size of 12 was required to achieve a power of 80% at a type I error rate of 0.05. A total of 50 male participants were recruited to complete this study, including three groups: (1) patients who underwent ACLR on their dominant limb (ACLR-D group, n = 17); (2) patients who underwent ACLR on their nondominant limb (ACLR-ND group, n = 16); (3) Healthy individuals matched for age, height, weight, and physical activity level to the ACLR patients, were selected as the control group (CON group, n = 17).

The patients with ACLR were recruited from Qilu Hospital of Shandong University, and the participants of CON group were recruited from Shandong Sport University. The inclusion criteria for this study were as follows: (1) aged 18–40 years; (2) Unilateral hamstring tendon reconstruction without combined meniscal medial collateral ligament injury; (3) hospital-assessed to meet criteria for return to sport; (4) 10–14 months after ACLR; (5) Willingness to return to sports (RTS) after ACLR; (6) both pre-injury ACLR patients and healthy athletes regularly participated in at least one physical activity daily; (7) Tegner Activity Scale ≥5. The exclusion criteria were as follows: (1) knee-related injury within 3 months; (2) previous other knee-related surgeries; (3) severe cardiovascular and neurological disease history; (4) visual impairment and intolerable associated organ disease. The study was approved by the Ethics Committee of Sports Science of Shandong Sports University (approval number: 2023004) and registered with the China Clinical Trial Registry (registration number: ChiCTR2300076299). All patients signed the informed consent form before participation.

### 2.2 Procedures

This cross-sectional study design was completed in the biomechanics laboratory of the Shandong Sport University. Participants were recruited between Oct. 2023 and May 2024. Before the biomechanical assessment, participants completed the International Knee Documentation Committee (IKDC) to assess knee function. Participants’ demographic information, surgery information, and dominant limb are shown in Table 1. Limb dominance was determined by which limb they were more accustomed to using when kicking a ball (Zumstein et al., 2022).

**TABLE 1:** Participant information (mean ± SD).

	ACLR-D	ACLR-ND	CON	One-way ANOVA/T-test	
	(n = 17)	(n = 16)	(n = 17)	F/t value	P-Value
Age (years)	24.1 ± 4.3	23.9 ± 1.7	23.4 ± 1.6	0.242^a^	0.786
Height (cm)	176.4 ± 5.1	175.9 ± 5.7	178.1 ± 6.8	0.601^a^	0.553
Weight (kg)	76.6 ± 9.4	72.7 ± 11.3	73.6 ± 15.4	0.475^a^	0.625
Dominant limb, right/left (n)	11/6	11/5	16/1	NA	NA
Postoperative duration (months)	12.1 ± 1.6	11.8 ± 1.6	NA	0.442^b^	0.662
IKDC (score)	87.2 ± 9.4	86.6 ± 6.4	NA	0.447^b^	0.658
Tegner Activity Scale (score)	6.9 ± 1.4	6.6 ± 1.4	6.8 ± 1.2	0.356^a^	0.703

IKDC, International Knee Documentation Committee; ACLR-D, anterior cruciate ligament reconstruction on dominant limb; ACLR-ND, anterior cruciate ligament reconstruction on nondominant limb; CON, control; NA, not available.

^a^ F-value for one-way ANOVA.

^b^ t-value for independent samples T-test.

**FIGURE 1:**
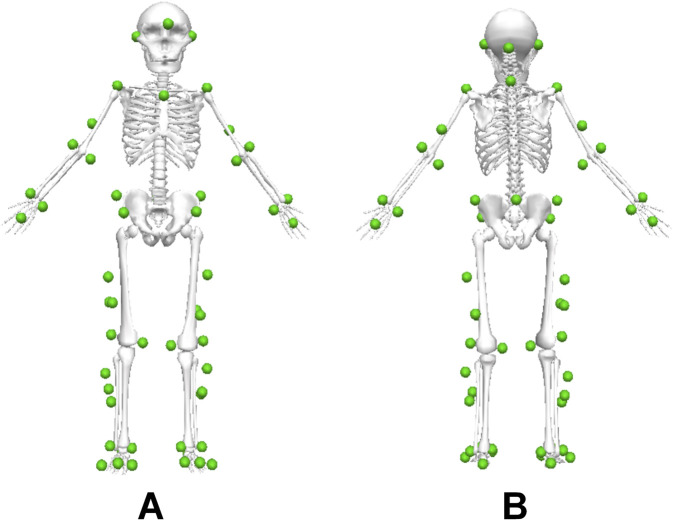
Reflective marker positions in the Visual 3D model. **(A)** view from the front. **(B)** view from behind.

Participants changed into spandex pants and t-shirts and wore running shoes provided by the laboratory. They were allowed to perform self-selected warm-up activities for 5 min before testing. Fifty-three reflective markers were placed on the head, trunk, and limbs, with three marker clusters placed on each thigh and shank (Figure 1). Twenty electrodes were placed bilaterally on the vastus medialis (VM), vastus lateralis (VL), rectus femoris (RF), biceps femoris (BF), and semitendinosus (ST) (He et al., 2022; Di Giminiani et al., 2023).

**FIGURE 2:**
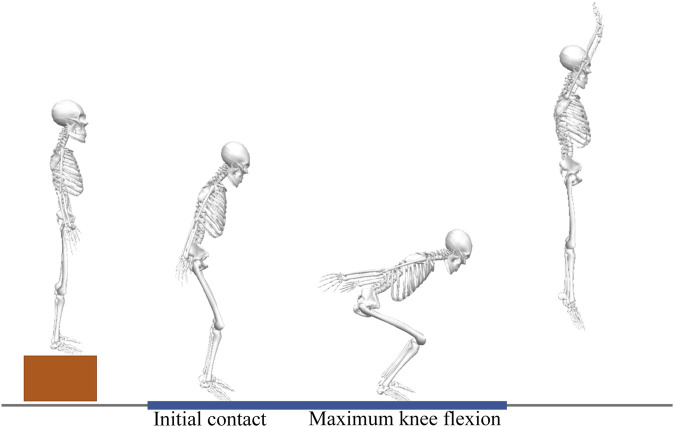
Drop vertical jump (DVJ) task. First landing-impact phase (time between initial contact with the ground and maximum knee flexion) of the DVJ task was analyzed.

Following a static calibration trial, participants conducted three successful trials of a DVJ task, along with three 5-s maximal voluntary isometric contraction (MVIC) tests for the quadriceps and hamstrings. For the DVJ task, participants were asked to jump forward from a 30 cm-high box onto force platforms, and immediately jump as high as possible (Baellow et al., 2020) (Figure 2). Participants landed on the two force plates with both feet respectively without falling, and all signals were collected which was considered as a successful trial. Participants were allowed to swing their arms as needed during jumps. The MVIC test for quadriceps were performed with participants in sitting with 60° of knee flexion, while hamstrings were performed with 30° of knee flexion in prone position (Kotsifaki et al., 2022b). Participants were given a 1-min rest between trials to reduce the effects of fatigue.

The three-dimensional positions of the reflective markers were captured using 12 infrared cameras at a sampling frequency of 200 Hz (Vicon Motion Systems Ltd., Oxford, United Kingdom). Bilateral ground reaction forces (GRF) data were collected using two force platforms (AMTI, Inc., Watertown, MA, United States) at a sampling frequency of 1,000 Hz. Electromyographic (EMG) signals were collected using a wireless surface EMG system (Noraxon, Arizona, United States) at a sampling frequency of 2000 Hz. The coordinate signals of markers and analog signals of GRF and EMG data collection were time synchronized using Nexus software (Vicon Motion Systems Ltd., Oxford, United Kingdom).

### 2.3 Data reduction

Raw marker coordinates and GRF data were filtered using a fourth-order, zero-phase Butterworth filter at a low-pass of 10 Hz (Kim et al., 2015) and 50 Hz (Teng et al., 2017), respectively. Knee joint angles were using a Cardan X-Y-Z sequence of rotations, defined as the angle between the distal and proximal segments (Kotsifaki et al., 2022b). Knee joint moments were computed using the inverse dynamics approach (Mausehund and Krosshaug, 2024). Posterior peak GRF (PPGRF) is the first peak of the posterior GRF (Dai et al., 2015), and peak vertical GRF (PVGRF) is the maximum vertical GRF in the first landing-impact phase (time between initial contact with the ground and maximum knee flexion). Posterior and vertical GRF and knee joint moments were normalized to body weight (kg).

Raw EMG signals for MVIC and dynamic tasks were filtered with a 20–500 Hz bandpass filter (Markström et al., 2022), and smoothed using a root-mean-square algorithm with a 50 milliseconds moving window (Frank et al., 2016). The integral of EMG (IEMG) signals for assessing muscle activity during the first landing-impact phase for each muscle was calculated using the following Equation 1 (Urbanek and Van Der Smagt, 2016; Baellow et al., 2020):

$$I\text{EMG}=\int_{t_{1}}^{t_{2}}\left|X\left(t\right)\right|dt$$ (1)

t_1_ is initial contact, t_2_ is maximum knee flexion, X(t) is the EMG signal. The mean time of the first landing-impact phase of the three DVJ tasks for each participant was used to calculate the IEMG in the MVIC task.

The dynamic IEMG data were normalized to the MVIC tests, therefore IEMG data were reported as %MVIC. All data processing was performed in Visual 3D software (C-Motion Inc., Germantown, United States).

### 2.4 Statistical analysis

Data normality was determined using the Shapiro-Wilk test. One-way ANOVAs or independent t-tests were used to compare differences in participants’ demographic information among groups. Two-way repeated-measures ANOVAs (limb × group) were performed for variables of interest to examine the main effects of limb (dominant vs. nondominant) and group (ACLR-D, ACLR-ND, and CON), as well as the interaction between limb and group. Paired and independent t-tests were used to *post hoc* tests to compare differences between limbs and groups, respectively, if no significant interaction effect was detected but significant main effects were detected. One-way ANOVAs were used to determine the effects of each independent variable on a given dependent variable if a significant interaction effect was detected. The significant level was set at α = 0.05. Partial η^2^ (η^2^_p_) was used to indicate the effect sizes of two-way ANOVAs for the main and interaction effects. The thresholds for η^2^_p_ were: 0.01–0.06 for small, 0.06–0.14 for medium, and greater than 0.14 for large effect sizes (Pierce et al., 2004). All data were statistics in SPSS 26.0 and presented as mean ± SDs.

## 3 Results

Significant limb × group interactions were observed for knee valgus angle (*p* = 0.019; η^2^_p_ = 0.172), knee extension moment (P < 0.001; η^2^_p_ = 0.318), and knee valgus moment (P = 0.027; η^2^_p_ = 0.142). *post hoc* tests demonstrated that the nonsurgical limbs of the ACLR-D and ACLR-ND groups had significantly greater knee valgus angle and knee extension moment compared to the surgical limbs, and the knee valgus moments of the nonsurgical limbs were greater than the surgical limbs in ACLR-ND group. For the nonsurgical limbs, the ACLR-ND group exhibited significantly greater knee valgus moments compared to the CON group. No any main effects or interactions were observed in knee flexion angle, knee external rotation angle, and knee internal rotation moment (Table 2).

**TABLE 2:** Knee joint angles and moments at PPGRF during the landing phase in drop vertical jump (DVJ) task (mean ± SD).

	ACLR-D		ACLR-ND		CON		P (η^2^_p_)		
	Nonsurgical	Surgical	Nonsurgical	Surgical	Dominant	Non-dominant	Limb	Group	Interaction
Knee flexion angle (°)	28.4 ± 8.6	30.1 ± 9.0	30.9 ± 8.1	31.6 ± 8.4	31.9 ± 10.0	32.4 ± 9.1	0.314 (0.022)	0.573 (0.023)	0.842 (0.007)
knee valgus angle^※^ (°)	−2.9 ± 2.2	−1.4 ± 0.9^a^	−3.1 ± 2.0	−1.5 ± 1.1^a^	−1.7 ± 1.1	−1.9 ± 1.0	-	-	0.019 (0.172)
knee external rotation angle^※^ (°)	−4.2 ± 3.0	−4.9 ± 4.7	−5.4 ± 3.9	−4.6 ± 3.6	−5.7 ± 4.6	−4.5 ± 3.8	0.519 (0.009)	0.881 (0.005)	0.485 (0.030)
knee extension moment (Nm/kg)	1.36 ± 0.46	1.19 ± 0.36^a^	1.67 ± 0.46^b^	1.21 ± 0.26^a^	1.31 ± 0.26	1.32 ± 0.31	-	-	<0.001 (0.318)
knee valgus moment^※^ (Nm/kg)	−0.14 ± 0.11	−0.13 ± 0.08	−0.25 ± 0.23^b^	−0.11 ± 0.07^a^	−0.11 ± 0.06	−0.12 ± 0.07	-	-	0.027 (0.142)
knee internal rotation moment (Nm/kg)	0.03 ± 0.11	0.02 ± 0.04	0.04 ± 0.09	0.02 ± 0.05	0.03 ± 0.06	0.02 ± 0.02	0.277 (0.025)	0.973 (0.001)	0.916 (0.004)

PPGRF, peak posterior ground reaction force; ACLR-D, anterior cruciate ligament reconstruction on dominant limb; ACLR-ND, anterior cruciate ligament reconstruction on nondominant limb; CON, control.

^※^ Knee valgus angle, knee external rotation angle and knee valgus moment were defined as negative numbers.

^a^ Significant difference within-group.

^b^ Significant difference compared with CON, group.

Significant limb × group interactions were observed for the muscle activation in VM (P = 0.007; η^2^_p_ = 0.203), RF (P = 0.007; η^2^_p_ = 0.195), and VL (P = 0.006; η^2^_p_ = 0.206). Post hoc tests demonstrated that the muscle activation of the surgical limbs on VM, RF, and VL in the ACLR patients and of the nonsurgical limbs on VM and VL in the ACLR-ND group was significantly lower than that in the CON group. The nonsurgical limbs of the ACLR-D group had significantly greater muscle activation in VM, RF, and VL compared to the surgical limbs (Table 3).

**TABLE 3:** Landing-impact time and muscle activation during the landing phase in drop vertical jump (DVJ) task (mean ± SD).

	ACLR-D		ACLR-ND		CON		P (η^2^_p_)		
	Nonsurgical	Surgical	Nonsurgical	Surgical	Dominant	Non-dominant	Limb	Group	Interaction
landing-impact time (s)	0.278 ± 0.048	0.274 ± 0.053	0.277 ± 0.050	0.281 ± 0.055	0.294 ± 0.052	0.296 ± 0.051	0.904 (0.001)	0.510 (0.028)	0.266 (0.055)
VM (%MVIC)	113.6 ± 66.7	64.3 ± 40.1^ab^	69.9 ± 35.6^a^	63.1 ± 36.5^a^	154.8 ± 43.9	170.5 ± 66.5	-	-	0.007 (0.203)
RF (%MVIC)	84.3 ± 62.0	45.3 ± 32.8^ab^	63.3 ± 26.0	42.8 ± 29.4^a^	100.5 ± 27.4	108.9 ± 43.1	-	-	0.007 (0.195)
VL (%MVIC)	94.5 ± 52.3	53.1 ± 34.4^ab^	58.4 ± 35.9^a^	59.5 ± 42.4^a^	132.2 ± 45.3	144.8 ± 51.3	-	-	0.006 (0.206)
BF (%MVIC)	15.9 ± 9.1^a^	13.3 ± 8.7^a^	10.9 ± 4.9^a^	15.9 ± 8.6^a^	30.3 ± 18.5	25.6 ± 7.9	0.682 (0.004)	<0.001 (0.398)	0.095 (0.101)
ST (%MVIC)	13.7 ± 8.1^a^	15.8 ± 9.0^ab^	11.9 ± 4.8^a^	20.0 ± 13.0^ab^	25.5 ± 8.2	30.6 ± 12.3^b^	0.002 (0.213)	<0.001 (0.382)	0.283 (0.058)

VM, vastus medialis; RF, rectus femoris; VL, vastus lateralis; BF, biceps femoris; ST, semitendinosus; MVIC, maximal voluntary isometric contraction; ACLR-D, anterior cruciate ligament reconstruction on dominant limb; ACLR-ND, anterior cruciate ligament reconstruction on nondominant limb; CON, control.

^a^ Significant difference compared with CON, group.

^b^ Significant difference within-group.

No significant limb × group interactions were found for the muscle activation in BF and ST, while a significant group effect was detected for both BF (P < 0.001; η^2^_p_ = 0.398) and ST (P < 0.001; η^2^_p_ = 0.382), as well as a main effect for limb in ST (P = 0.002; η^2^_p_ = 0.213). Post hoc tests demonstrated that the BF and ST activation in ACLR patients were significantly smaller compared to the CON group. Additionally, the nonsurgical limbs of the ACLR patients exhibited significantly smaller ST activation compared to the surgical limbs. No significant differences between or within groups were detected in the landing-impact time during the DVJ task (Table 3).

No significant limb × group interactions were detected on any muscle activation in MVIC tasks. Significant group main effects were observed only in muscle activation of VL (P = 0.016; η^2^_p_ = 0.161) and BF (P = 0.003; η^2^_p_ = 0.219) in the MVIC task. Post hoc tests demonstrated that ACLR patients had significantly lower activation of both VL and BF in bilateral limbs than the CON group (Table 4).

**TABLE 4:** Muscle activation during the maximal voluntary isometric contraction (MVIC) task (mean ± SD).

	ACLR-D		ACLR-ND		CON		P (η^2^_p_)		
	Nonsurgical	Surgical	Nonsurgical	Surgical	Dominant	Non-dominant	Limb	Group	Interaction
VM_MVIC (μv·s)	54.0 ± 12.4	52.2 ± 11.5	60.0 ± 14.8	53.0 ± 19.4	66.3 ± 20.6	63.3 ± 25.4	0.106 (0.055)	0.101 (0.093)	0.657 (0.018)
RF_MVIC (μv·s)	62.4 ± 9.3	60.2 ± 9.1	67.1 ± 18.5	63.1 ± 15.4	68.5 ± 14.9	67.2 ± 16.1	0.189 (0.036)	0.331 (0.046)	0.852 (0.007)
VL_MVIC (μv·s)	55.3 ± 17.6^a^	57.8 ± 16.0^a^	57.4 ± 14.1^a^	55.9 ± 8.0^a^	67.7 ± 8.7	66.3 ± 8.7	0.946 (0.001)	0.016 (0.161)	0.475 (0.031)
BF_MVIC (μv·s)	65.4 ± 9.6^a^	64.9 ± 13.8^a^	66.8 ± 6.2^a^	68.4 ± 8.1^a^	78.8 ± 21.5	79.8 ± 14.1	0.659 (0.004)	0.003 (0.219)	0.854 (0.007)
ST_MVIC (μv·s)	78.3 ± 13.6	80.0 ± 12.2	79.9 ± 16.0	77.4 ± 8.7	83.3 ± 15.0	81.3 ± 16.4	0.687 (0.003)	0.601 (0.021)	0.714 (0.014)

VM, vastus medialis; RF, rectus femoris; VL, vastus lateralis; BF, biceps femoris; ST, semitendinosus; MVIC, maximal voluntary isometric contraction; ACLR-D, anterior cruciate ligament reconstruction on dominant limb; ACLR-ND, anterior cruciate ligament reconstruction on nondominant limb; CON, control.

^a^ Significant difference compared with CON group.

**FIGURE 3:**
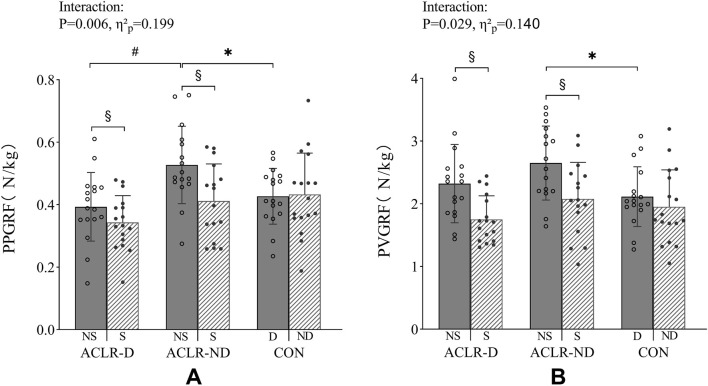
PPGRF **(A)** and PVGRF **(B)** in the first landing-impact phase of the DVJ task. PPGRF, peak posterior ground reaction force. PVGRF, peak vertical ground reaction force. NS, nonsurgical limb. S, surgical limb. D, dominant limb. ND, nondominant limb. ACLR-D, anterior cruciate ligament reconstruction on dominant limb. ACLR-ND, anterior cruciate ligament reconstruction on nondominant limb. CON, control. * Significant difference compared with CON group. # Significant difference compared with ACLR-ND group. § Significant difference within-group.

Significant limb × group interactions were observed for PPGRF (P = 0.006; η^2^_p_ = 0.199) and PVGRF (P = 0.029; η^2^_p_ = 0.140). Post hoc tests demonstrated that the nonsurgical limbs of the ACLR-D and ACLR-ND groups had significantly greater PPGRF and PVGRF compared to the surgical limbs. For the nonsurgical limbs, the ACLR-ND group exhibited significantly greater PPGRF and PVGRF compared to the CON group. Additionally, the ACLR-ND group showed greater PPGRF in the nonsurgical limbs compared to the ACLR-D group (Figure 3).

## 4 Discussion

The results of this study partially support our first hypothesis, indicating that the nonsurgical limbs exhibited greater GRFs and knee joint moments compared to the surgical limbs in both ACLR-D and ACLR-ND groups, with the exception of quadriceps and hamstring muscle activation. The results of this study demonstrated that the nonsurgical limbs of ACLR patients exhibited greater PPGRF and PVGRF compared to surgical limbs in the DVJ task. These findings were consistent with previous studies that have shown ACLR patients reduce the weight bearing of the surgical limbs during exercises (Song et al., 2023; Baumgart et al., 2017) due to quadriceps inhibition and weakness (Palmieri-Smith et al., 2019; Pietrosimone et al., 2022). This self-protective mechanism (Baumgart et al., 2017) reduces the impact of GRF on the surgical knee, potentially mitigating the risk of further injury. The current study results suggested a possible change in the movement pattern and neuromuscular control strategy used by ACLR patients when performing the DVJ, which may be characterized by an altered landing strategy. Despite the synchronous movement of both limbs during the DVJ, ACLR patients actively shift their center of gravity towards the nonsurgical limbs, resulting in greater GRF being absorbed by the nonsurgical limbs, which may contribute to an increased risk of ACL injury. Previous studies have shown that greater GRF can increase tibiofemoral joint compression forces, which is a known risk factor for ACL injury (Meyer et al., 2008; Boden et al., 2010). Therefore, these findings suggested that patients in both the ACLR-D and ACLR-ND groups may be at an increased risk of ACL injury in their nonsurgical limbs compared to the surgical limbs during DVJ task, potentially due to the altered movement patterns and neuromuscular control strategies.

The current study revealed no significant differences in knee angles and moments at PPGRF between the dominant and nondominant limbs of healthy individuals during the DVJ task. This may suggest that ACLR patients have no inherent differences in the bilateral limbs prior to the ACL injury. Conversely, our results indicated that ACLR patients demonstrated greater knee valgus angles and extension moments in their nonsurgical limbs compared to their surgical limbs, as well as the ACLR-ND patients also exhibited greater knee valgus moments in their nonsurgical limbs. This is consistent with previous studies that have reported reduced surgical knee loading in patients with ACLR (Sritharan et al., 2020; Kotsifaki et al., 2022b; Bühl et al., 2023). The reason for these results may be an adaptive change in the landing strategy of ACLR patients. ACLR patients may rely more heavily on their nonsurgical limbs due to decreased VM, VL and RF muscle strength, impaired knee proprioception, and reduced stability in the surgical limbs (Howells et al., 2011; Arumugam et al., 2021), which can lead to an adaptive change in their landing strategy. This also validated that asymmetry of knee moments is associated with asymmetric GRF, which can lead to altered movement patterns and increased risk of injury (Dai et al., 2014). As results of these adaptive changes in landing strategy, ACLR patients may be at an increased risk of ACL injury in their nonsurgical limbs, particularly during dynamic movements that involve landing.

The results of this study partially support our second hypothesis that the nonsurgical limbs in the ACLR-ND patients exhibited greater knee extension and valgus moments, as well as greater GRFs compared to CON group, which may contribute to an increased risk of ACL injury. Furthermore, in the nonsurgical limbs, the ACLR-ND patients demonstrated greater PPGRF compared to the ACLR-D patients, as well as greater GRFs, and greater knee valgus and extension moments compared to the CON group. These differences did not exist between the ACLR-D and CON groups. These were similar to previous studies on single-leg jump (Mohammadi et al., 2013), side cut (Warathanagasame et al., 2023), and stair walking (Zabala et al., 2013) tasks. However, in contrast with our results, neither Rush et al. (2024) nor Chen et al. (2024) observed greater GRF and knee extension and valgus moments in the nonsurgical limbs compared to the healthy individuals in single-leg jump or DVJ tasks. The inconsistent results may be attributed to the fact that these studies did not account for the potential influence of limb dominance on movement patterns after ACLR. Notably, the nonsurgical limb was the dominant limb in the ACLR-ND patients in the current study, which may have influenced their movement patterns and neuromuscular control strategies during the landing task. Compared to ACLR-D, the ACLR-ND patients may have been more inclined to use a protective pattern, characterized by increased knee extension and valgus moments, on the nonsurgical limbs during the landing phase, and felt more confident with aggressive landings. However, for ACLR-ND patients, this protective movement pattern may have unintended consequences, as the increased knee loading on the nonsurgical limbs may actually increase the risk of ACL injury, rather than reducing it.

Contrary to our initial hypothesis, no significant differences in muscle activation levels of the quadriceps and hamstrings were observed in the nonsurgical limbs between ACLR-ND and ACLR-D patients. However, our study revealed that muscle activation in the VM and VL of the nonsurgical limbs was significantly decreased during DVJ task in ACLR-ND patients compared to CON group, in addition to reduced activation of the quadriceps and hamstrings of the surgical limbs. Additionally, muscle activation in BF and ST of the nonsurgical limbs was significantly lower during DVJ task in both ACLR-ND and ACLR-D patients compared to CON group. These results were consistent with literatures, which also reported lower bilateral quadriceps and hamstring activation in ACLR compared to healthy controls (Alanazi et al., 2020; Einarsson et al., 2021). This may be attributed to reduced quadriceps and hamstring muscle strength and neuromuscular inhibition (Palmieri-Smith et al., 2019; Pietrosimone et al., 2022). Additionally, a recent study reported that increased quadriceps and hamstring activation was associated with reduced knee flexion angle (Malfait et al., 2016). Therefore, ACLR patients may attempt to obtain a greater knee flexion angle to reduce impact of GRF by reducing bilateral muscle activation. Lower quadriceps and hamstring activation was associated with reduced dynamic knee stability, which may be a contributing factor to the increased risk of ACL injury (Ortiz et al., 2014; Palmieri-Smith et al., 2019; Wang et al., 2023). These results combined together suggest that abnormal quadriceps and hamstring activation in ACLR-ND patients is associated with an increased risk of ACL injury. In summary, our study revealed significant differences in quadriceps and hamstring activation levels between dominant and nondominant limbs in ACLR patients. These findings emphasized the need for personalized rehabilitation programs that take into account limb dominance to optimize outcomes and reduce the risk of further injury in ACLR patients.

There are several limitations in our study. Firstly, all participants were male, and since gender differences in knee valgus angles, and GRFs during landing (Seymore et al., 2019; Peebles et al., 2020) may affect the applicability of our results to females, future studies should investigate the effects of limb dominance on biomechanics in female ACLR patients. Secondly, we only analyzed ACLR patients with autologous hamstring grafts. Since there is an effect of different graft types on knee biomechanics (Wang et al., 2018; Yang et al., 2020), further studies in patients with other graft types are needed. Third, we did not collect muscle strength, proprioception from the participants. Previous studies have indicated that muscle strength, proprioception affect knee function and athletic performance (Ma et al., 2022; Chang et al., 2024). Future studies should investigate the effect of limb dominance on functional outcomes. Fourth, we only investigated biomechanical characteristics in the DVJ task, and future studies should investigate the effects of limb dominance on biomechanics during various movement tasks, including single-leg jumps and side-cutting maneuvers. Fifth, we conducted a cross-sectional analysis, which did not allow us to examine the longitudinal effects of limb dominance on biomechanics after ACLR. The long-term effects of limb dominance on biomechanics after ACLR remain unclear and warrant further investigation.

## 5 Conclusion

The nonsurgical limbs of ACLR patients may suffer an increased risk of ACL injury due to altered landing mechanics and neuromuscular control strategies compared to the surgical limbs. Additionally, limb dominance influences movement patterns and neuromuscular control during DVJ task, the nonsurgical limbs of the ACLR-ND might be at higher risk of ACL injury compared to the ACLR-D group. Given that limb dominance affects movement patterns, the impact of limb dominance should be considered in the rehabilitation of ACLR patients for better return to sport.

## Data Availability

The original contributions presented in the study are included in the article/supplementary material, further inquiries can be directed to the corresponding authors.
